# *QuickStats*: Percentage of Residential Care Communities* that Use Electronic Health Records,^†^ by Community Bed Size — United States, 2016 and 2020^§^

**DOI:** 10.15585/mmwr.mm7139a7

**Published:** 2022-09-30

**Figure:**
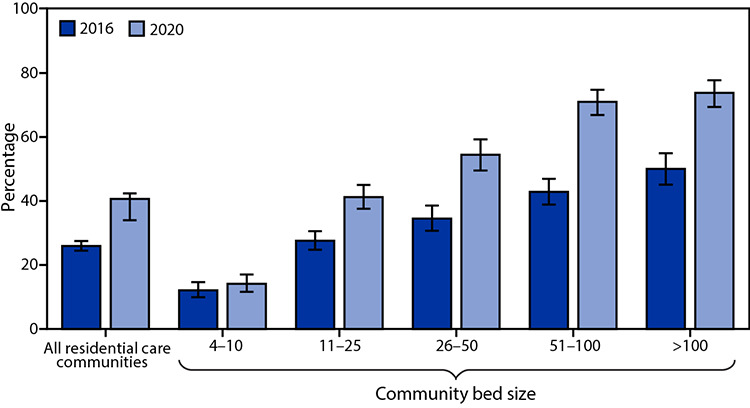
From 2016 to 2020, the percentage of residential care communities using electronic health records increased from 26% to 41%. The percentage using electronic health records increased from 28% to 41% for 11–25 bed communities, 35% to 54% for 26–50 bed communities, 43% to 71% for 51–100 bed communities, and 50% to 74% for more than 100 bed communities. The change (from 12% to 14%) was not significant for 4–10 bed communities.

**Source:** National Post-acute and Long-term Care Study, 2016 and 2020 data. https://www.cdc.gov/nchs/npals/questionnaires.htm

